# Reducing Inappropriate Surgical Referrals Through Patient Assessment Algorithm Utilising Early Imaging: A Closed-Loop Audit

**DOI:** 10.7759/cureus.95123

**Published:** 2025-10-22

**Authors:** Anil Kumar, Navjot S Dhillon, Swetha Bunga, Meghana Taggarsi, Sunetra Chatterjee, Sajal Rai

**Affiliations:** 1 Colorectal and General Surgery, Stepping Hill Hospital, Stockport NHS Foundation Trust, Stockport, GBR; 2 Colorectal and General Surgery, Pilgrim Hospital, United Lincolnshire Teaching Hospitals NHS Trust, Boston, GBR; 3 Paediatric Surgery, Royal Victoria Infirmary, Newcastle upon Tyne Hospitals NHS Foundation Trust, Newcastle upon Tyne, GBR; 4 Colorectal and General Surgery, Peterborough City Hospital, North West Anglia NHS Foundation Trust, Peterborough, GBR

**Keywords:** acute abdominal pain pathway, closed-loop audit, emergency department, general surgery, nhs efficiency, patient flow optimisation, referral appropriateness, resource utilisation

## Abstract

Introduction: Emergency department (ED) referrals to specialist services are integral to patient management within the National Health Service (NHS). However, rising pressures, the lack of standardised referral protocols, incomplete assessments and time constraints often result in inappropriate referrals, compromising patient care and resource utilisation. This study evaluated the appropriateness of referrals from the ED to general surgery (GS) through a two-cycle audit with a targeted intervention, acute abdominal pain pathway, incorporating early computed tomography (CT) imaging.

Methods: A closed-loop audit was conducted at Stepping Hill Hospital, Stockport, the United Kingdom. All ED-to-GS referrals over one month in July 2020 (Cycle 1) and July 2024 (Cycle 2) were included. Data were collected on assessment completeness, investigations performed, preliminary treatment, the grade of accepting surgical doctor and alignment between ED and surgical diagnoses. Inappropriate referrals were defined as those with discordant diagnoses or requiring onward referral to another speciality post-GS assessment. Following Cycle 1, an acute abdominal pain pathway emphasising early CT use was implemented. Data were analysed using descriptive statistics, chi-square tests and effect size calculations.

Results: In Cycle 1, 111 patients were referred to GS, 86 (77.5%) of whom had abdominal pain. Out of the 86, 34 (39.6%) lacked blood investigations, 53 (61.6%) had no basic radiology such as chest X-ray (CXR) or abdominal X-ray (AXR), 18 (21.9%) received no preliminary treatment and 24 (27.9%) were deemed inappropriate referrals. Following the intervention, Cycle 2 (n=72) showed marked improvements: referrals without blood tests decreased to five (20%) (p=0.001), those without treatment to three (4.2%) (p=0.001) and inappropriate referrals to seven (9.7%) (p=0.004), reflecting a 65.2% relative risk reduction.

Discussion: The findings underscore the critical role of structured referral pathways and early imaging in improving ED-to-GS referral quality. Incorporating CT at the point of initial assessment reduced inappropriate referrals, optimised resource allocation and improved patient flow, consistent with existing literature on CT's diagnostic accuracy and impact on reducing unnecessary admissions. This intervention demonstrates a pragmatic, scalable solution to ED pressures, enhancing both clinical efficiency and patient outcomes while supporting NHS objectives of safe, cost-effective care.

Conclusion: The implementation of an acute abdominal pain pathway with early CT imaging significantly improved the appropriateness of surgical referrals from ED, reduced inefficiencies and enhanced overall patient management. The model is readily generalisable and has the potential for widespread adoption to address systemic challenges, ensuring high standards of care amidst increasing healthcare demands.

## Introduction

Every year, there are around 26.8 million attendances in the emergency department (ED) in the United Kingdom's National Health Service (NHS). Out of these, around 13,200 are admitted each day to be referred to specialist services and departments for further management [1]. In principle, this process should be seamless, with patient care at its core, but the reality is far from it, as significant gaps exist between the design of this pathway and its delivery.

The four-hour performance metric is widely used for waiting time in the ED. This is the time taken from the arrival of a patient to their discharge, admission or transfer to a different hospital. Nationally, the benchmark is to keep it under 95% for the patient to be seen, treated and either discharged or admitted. With rising ED pressures in the NHS, the situation has become worse. It is not surprising that as high as 17% of these speciality referrals are made between three hours and 50 minutes and four hours [2].

ED is not just a place for triaging patients for referrals to different specialities but rather a place to assess, initiate treatment and, in some cases, offer definitive management. Optimal interspeciality referral necessitates critical information, such as the presenting complaints, initial assessment findings, observations and pertinent clinical details [3,4]. There must also be a primary set of investigations performed to establish the appropriateness of the referral and better aid the diagnosis, management and quality of care.

Within the broader context of increasing demand and operational pressures, the absence of standardised referral protocols and clearly defined care pathways in many centres further hinders the timely and appropriate escalation of patients to the correct speciality [5]. This problem is compounded by incomplete or unreliable patient histories, non-specific clinical presentations, limited practitioner experience, time constraints, workforce shortages and concerns over potential medicolegal repercussions. Considering all the factors together, they create a high-pressure clinical environment leading to inefficiency in operation and contributing to the frustration on both sides of the process.

This study was aimed at investigating the appropriateness of referrals from the ED to general surgery (GS) through a structured two-cycle audit with a targeted intervention between the cycles. The intervention was the introduction of a simplified patient assessment tool in the form of an acute abdominal pain pathway that included the use of an early computed tomography (CT) scan to evaluate the patients and formulate an appropriate treatment plan. The main objective of the audit was to find out if the number of inappropriate referrals to GS from ED could be reduced by the introduction of an assessment pathway with an overarching aim to improve patient care and optimise the utilisation of hospital resources.

## Materials and methods

The closed-loop audit was conducted at Stepping Hill Hospital, Stockport NHS Foundation Trust, Stockport, the United Kingdom. The audit was registered with the Trust's audit department. The first cycle of the audit was conducted over a month in July 2020.

The inclusion criteria were patients referred from the ED to GS, irrespective of their age. Patients referred from a general practitioner (GP) to the ED, who were subsequently referred to GS, were also included in the study. The exclusion criteria were children less than five years old, as this cohort of surgical patients was directly referred from the ED to tertiary centres.

Data were collected with regard to assessment in the ED, the availability of initial investigations such as bloods, chest X-ray (CXR)/abdominal X-ray (AXR), preliminary treatment, the grade of surgical doctor accepting the referrals, the clinical stability of patients before transfer to surgical wards and correlation between diagnosis at referral and final diagnosis on assessment by surgical doctors. The referrals were deemed inappropriate if there was a discrepancy between the initial referral diagnosis and final diagnosis on assessment by surgical doctors or if the patient had to be referred to a different speciality following surgical review.

**Figure 1:**
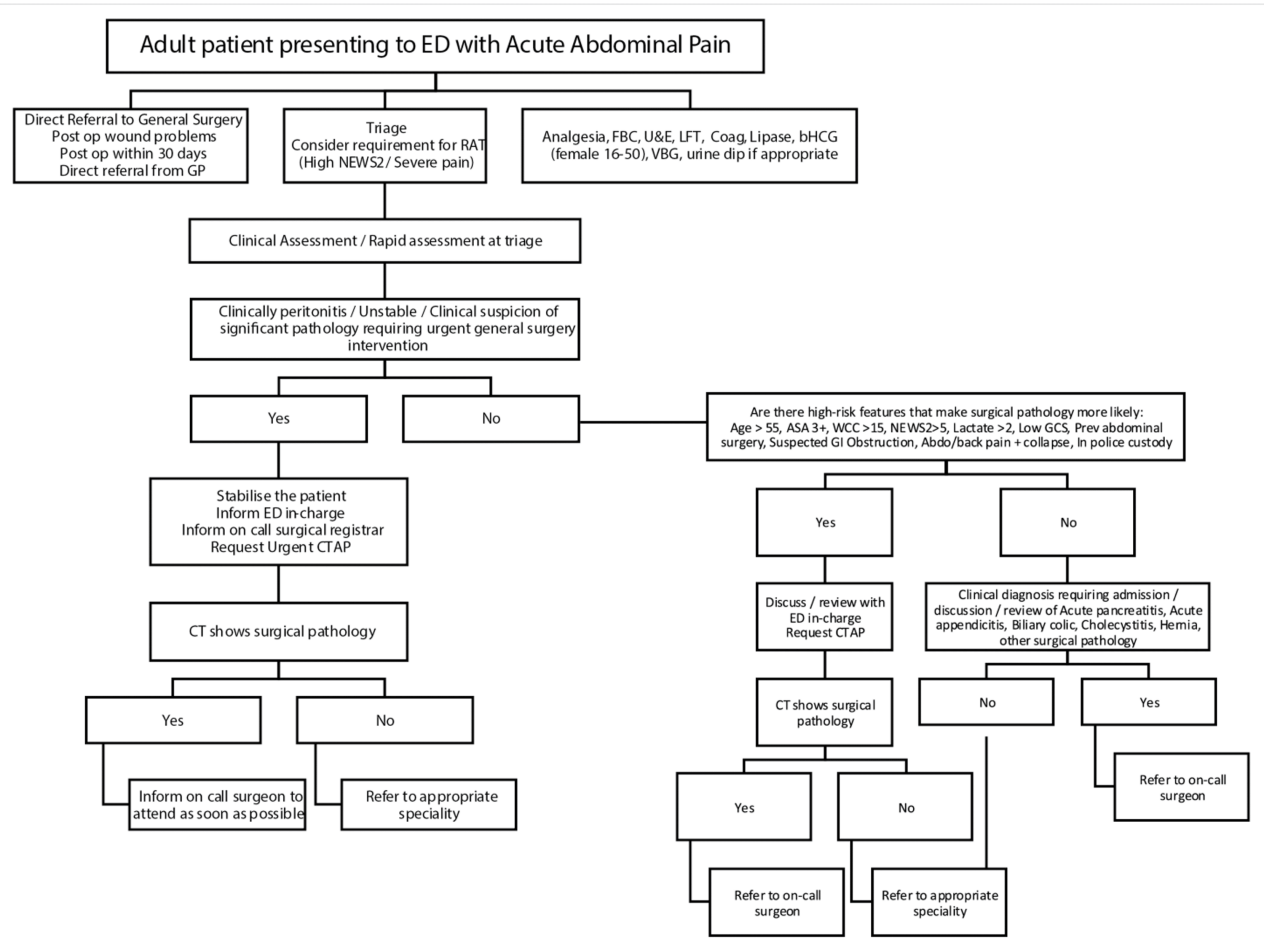
Acute Abdominal Pain Pathway Acute abdominal pain pathway developed for adult patients presenting to the ED to guide their management ED, emergency department; GP, general practitioner; ASA, American Society of Anesthesiologists; GCS, Glasgow Coma Scale; CT, computed tomography; U&E, urea and electrolytes; LFT, liver function test; RAT, rapid assessment at triage; NEWS2, National Early Warning Score 2; FBC, full blood count; bHCG, beta-human chorionic gonadotropin; VBG, venous blood gas; CTAP, CT of the abdomen and pelvis; WCC, white cell count

The results of the first cycle were presented and discussed in the departmental audit meeting, and an acute abdominal pain pathway (Figure 1) was devised to manage the patient flow more appropriately.

This intervention was followed up with a second cycle to evaluate the impact. This was conducted, in a similar way, over one month in July 2024. Data were collected and analysed based on the data characteristics of the first cycle. The findings were then compared and discussed in the audit meeting. The pathway was then adopted as standard in the hospital with plans to reconduct the audit at regular intervals to assess the efficacy and identify areas of further improvement.

Data were analysed using descriptive and inferential statistical methods. Categorical variables were summarised as numbers and percentages. Associations between categorical variables were assessed using the chi-square test. Relative risk reduction (RRR) and absolute risk reduction (ARR) were calculated to quantify the effect size, where appropriate. A p-value of <0.05 was considered statistically significant.

## Results

For a surgical referral from the ED, routine blood tests include complete blood count, C-reactive protein (CRP), urea and electrolytes (U&E), liver function test (LFT), amylase/lipase, urine dip, pregnancy test for women and blood gas (arterial or venous). In the first cycle, a total of 111 patients were referred to GS by the ED.

Seventy-two (64.9%) out of 111 referrals were discussed and accepted by the surgical registrar, six (5.4%) were referred to a senior house officer (SHO) and in 33 (29.7%) patients, no documentation was found regarding discussion/acceptance of referrals. Out of these, 24 (21.6%) were deemed to be inappropriate referrals based on adequate surgical assessment, the review of blood test and correlation with ED documentation and referral and were subsequently referred to other specialities, mainly to gynaecology, urology and medicine, or discharged home without the need for surgical intervention or follow-up. An important area of interest was identified, as all the inappropriate referrals were referred to GS as some form of abdominal pain. A total of 86 (77.5%) of the 111 patients were referred for abdominal pain. Thus, 27.9% (24/86) were inappropriate with respect to abdominal pain referrals.

On further sub-analysing the 86 patients referred with abdominal pain, we found that at the time of referral, blood investigations were available in only 52 (60.4%) patients, while 34 (39.6%) patients had no blood investigations. Basic radiological investigations such as chest X-ray (CXR) and/or abdominal X-ray (AXR) were not done in 53 (61.6%) patients. While 68 (79.1%) patients had some form of preliminary treatment initiated in the ED, 18 (21.9%) patients had no preliminary treatment, such as analgesics, intravenous fluids or antibiotics when required, given before surgical referral. We found that despite clinical evaluation, 61 (70.1%) required a computed tomography (CT) scan for assessment.

Five (20.8%) patients out of the 24 inappropriate referrals did not have any blood investigation done at the time of referral. This could have been prevented if routine blood investigations had been done and reviewed. Amongst the inappropriately referred patients, three (12.5%) referrals were accepted by the SHO and 14 (58.3%) by the registrar. Seven (29.2%) patients were referred inappropriately without even discussion with the on-call team.

Based on the first cycle findings, an acute abdominal pain pathway was introduced, incorporating the early use of CT imaging as a key tool for prompt and accurate diagnosis and more appropriate patient flow. The flowchart depicting the pathway was circulated amongst the healthcare professionals, both in the ED and amongst the GS team. Printouts were also placed on the ED notice board for easy reference. During the second cycle, a total of 95 patients were referred to GS by ED. Forty-one (43.2%) out of 95 referrals were discussed and accepted by the surgical registrar, 19 (20%) were referred to the SHO and in 35 (36.8%) patients, no documentation was found regarding the discussion/acceptance of referrals. Out of these, 72 (75.8%) were referred with abdominal pain. Since the pathway was focused on managing patients with abdominal pain, these were further sub-analysed.

On the sub-analysis of the 72 patients, three (4.2%) had no blood investigations before referral. As the second cycle involved doing a CT scan for acute abdominal pathologies, if appropriate, the need for routine CXR/AXR was reduced significantly. Thirty-eight (52.3%) out of 72 patients had a CT scan done in the ED as part of the acute abdominal pathway. Five (6.9%) did not receive preliminary treatment. Seven out of 72 (9.7%) referrals to GS were found to be inappropriate in the second cycle. Of the inappropriate referrals, three (42.9%) were accepted by the registrar, three (42.9%) were accepted by the SHO, and one (14.2%) occurred without prior discussion with the on-call team.

**Figure 2:**
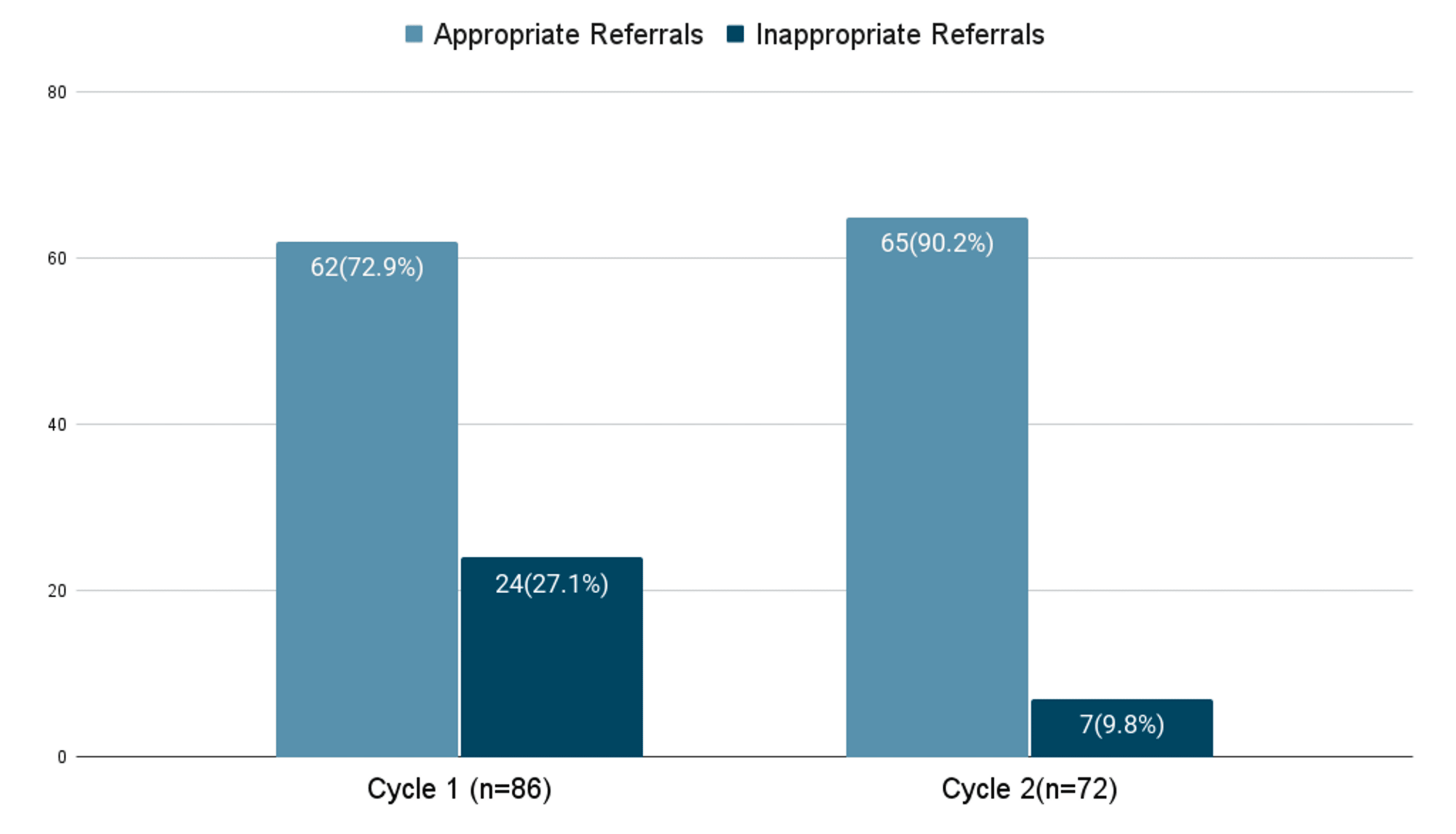
Number of Inappropriate Abdominal Pain Referrals in Cycle 1 Versus Cycle 2 Comparative graph showing the number of appropriate and inappropriate abdominal pain referrals from the ED to GS in Cycle 1 versus Cycle 2

**Figure 3:**
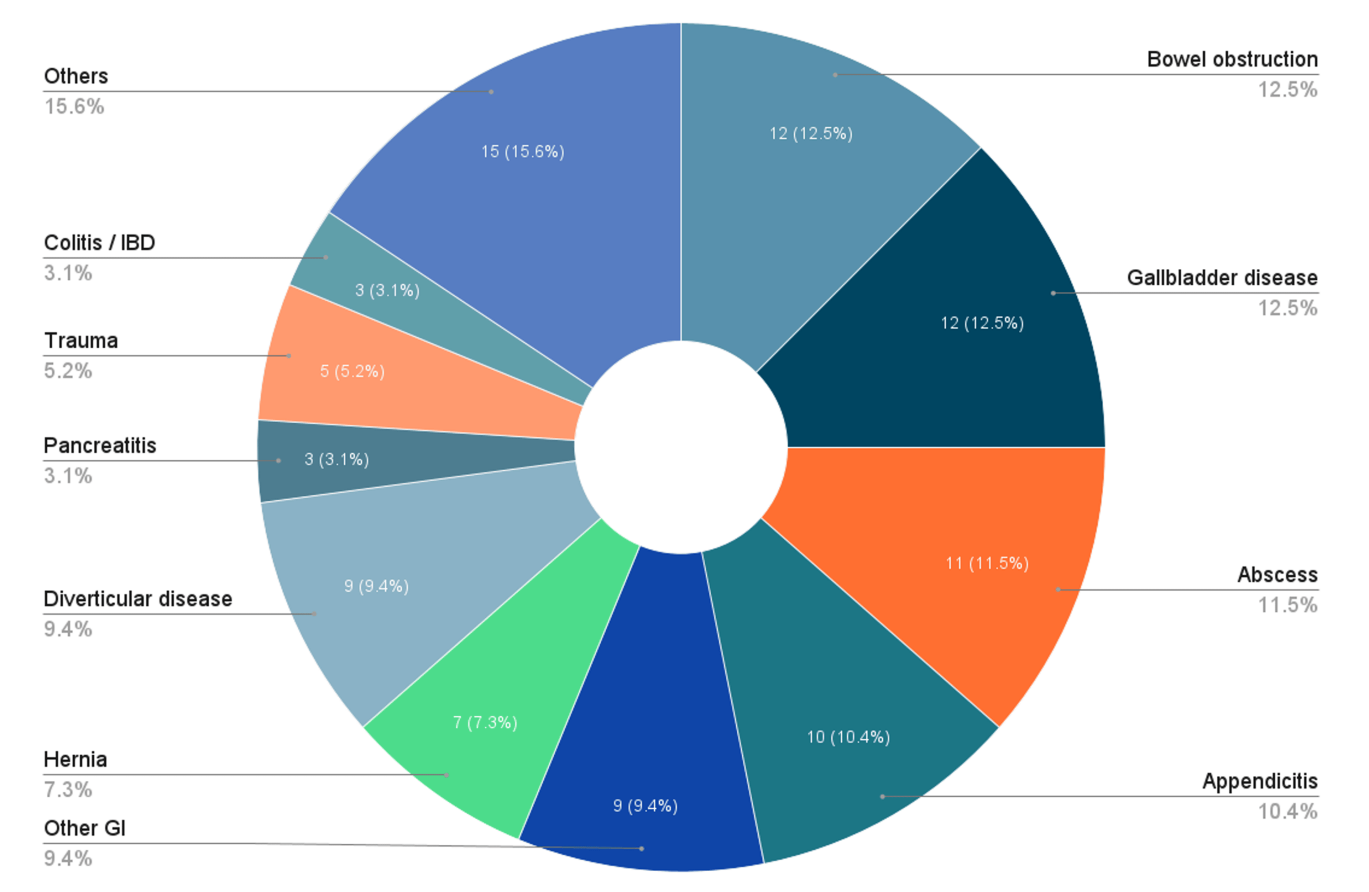
Distribution of Diseases as per Surgical Assessment in Cycle 1 (N=111) Pie chart showing the distribution of presentations as per assessment by the surgical team in Cycle 1 IBD: irritable bowel disease

**Figure 4:**
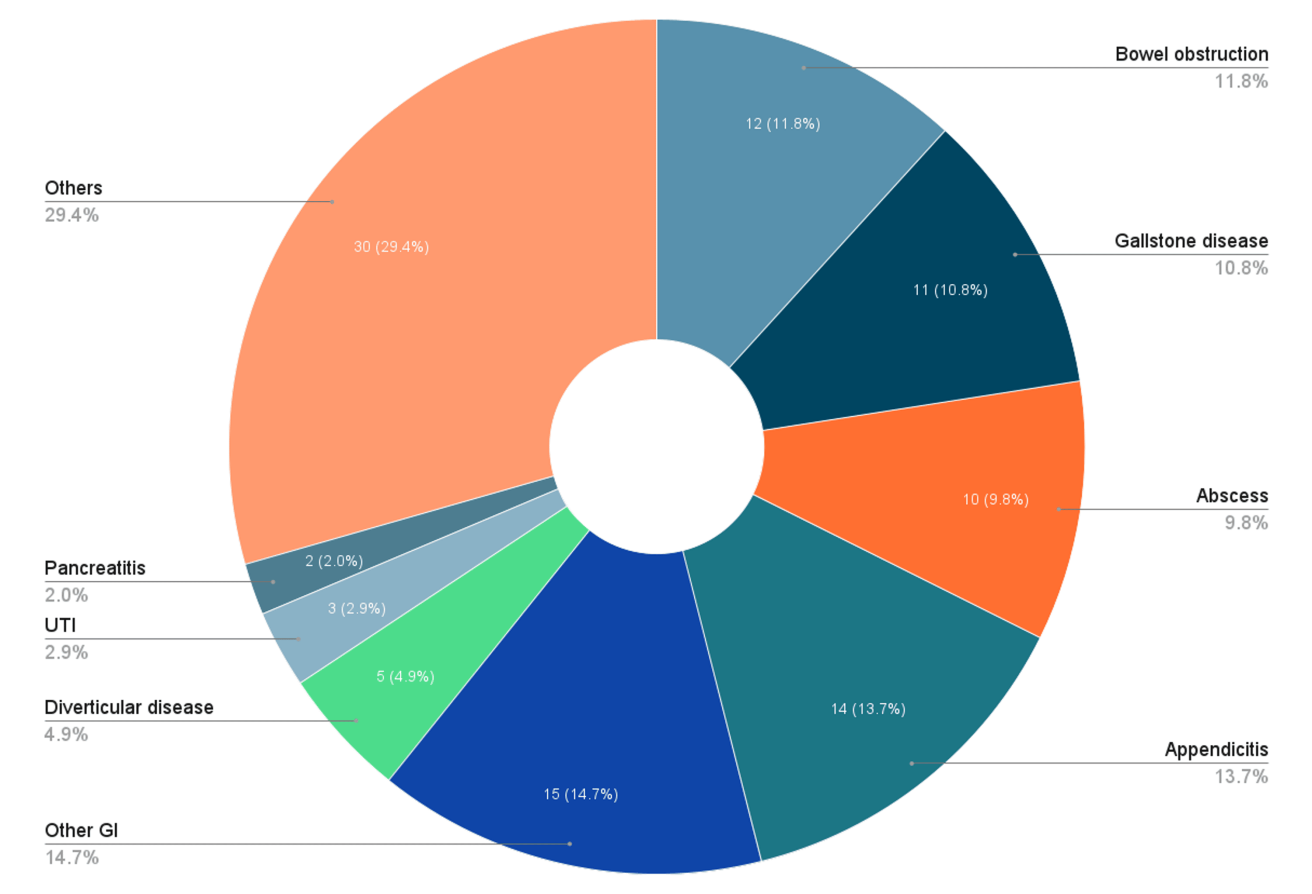
Distribution of Diseases as per Surgical Assessment in Cycle 2 (N=95) Pie chart showing the distribution of presentations as per assessment by the surgical team in Cycle 2

The proportion of patients referred without blood tests significantly decreased from 39.6% (34/86) in Cycle 1 to 4.2% (3/72) in Cycle 2 (χ², p=0.001). The proportion of patients without preliminary treatment decreased from 21.9% (18/86) to 6.9% (5/72), showing a statistically significant downward trend (χ², p=0.013). Notably, inappropriate referrals decreased significantly from 27.9% (24/86) to 9.7% (7/72) (χ² p=0.004) (Figure 2). Thus, the ARR is 18.2%, and the RRR is 65.2%. Overall, the introduction of the acute abdominal pathway with the routine use of early CT scan was associated with marked and statistically significant improvement in referral quality. Figure 3 and Figure 4 show the distribution of diseases as per the surgical assessment in the first and second cycles of the audit, respectively.

## Discussion

The NHS is the cornerstone of public healthcare, and it is incumbent upon all staff to deliver care of the highest standard while ensuring the optimal utilisation of finite resources. In England, government funding for the NHS totals approximately £171 billion annually, of which nearly 49% is spent on staff salaries [6,7]. Time and workforce are limited, and transferring responsibilities between departments merely to satisfy arbitrary administrative deadlines undermines efficiency and standards of care. While numerous systemic challenges persist, even modest, targeted interventions can produce significant improvements in service delivery.

The percentage of patients with a wait time of longer than four hours has risen significantly from around 4% to over 40% in the 10 years between April 2013 and April 2023 [1,8]. The target of this number to be kept below 5% is very difficult to meet and creates pressure on the staff to skip steps. While the government, in 2024, has planned additional funding for the worst-affected areas, it will take time to make a meaningful difference [9,10]. It is thus imperative that we develop ways to utilise resources better, leading to reduced costs while still upholding the highest standards of care [11].

Over time, CT scan has become an accessible imaging modality, which has made it integral to the assessment of patients presenting with suspected surgical pathologies [12]. CT has been shown to have a diagnostic accuracy of 82%-93%. Due to this, CT scans not only guide the treatment strategies but also help in excluding nonsurgical conditions [13]. Its utilisation has been associated with a 17% reduction in hospital admissions and a decline in the need for immediate surgical intervention from 13% to 5% [14]. These findings underscore the pivotal and time-sensitive role of CT in surgical decision-making. However, when the initiation of CT imaging is delayed until after speciality review, valuable time is lost, potentially postponing definitive management and adversely affecting patient outcomes.

From the GS perspective, this becomes particularly problematic when patients with medical conditions are misdiagnosed or inappropriately assessed and subsequently referred to GS. Such misdirection can compromise the quality and timeliness of patient care. The situation is complicated by the 'one-way referral' practice in many emergency departments, whereby once a patient is referred, whether appropriately or not, the receiving speciality assumes responsibility for assessment and, if necessary, onward referral. This approach can result in significant delays, especially if the initial referral is incorrect, as patients may then be admitted under an inappropriate speciality. In the eventuality that the referral to the appropriate speciality is declined, those patients then remain under the surgical team, leading to the improper utilisation of resources. This also diverts resources from those who would benefit from being treated by the appropriate speciality. Ultimately, such inefficiencies compromise both patient outcomes and the core principles underpinning the NHS [15].

A recent study done by Marchese et al. evaluating the referral process from the ED to GS found that 19% of all surgical referrals from the ED were inappropriate [5]. Similar findings were reported by Croft et al. [3]. Urso-Baiarda investigated the relationship between timing and the appropriateness of acute plastic surgery referrals and found that approximately one-third of patients were referred inappropriately. Interestingly, the study noted that inappropriate referrals were associated with shorter referral times. The author suggested that, to meet a four-hour waiting target with 98% compliance, patients must often be seen within an hour on average, necessitating even faster assessment for some to compensate for delays elsewhere. Consequently, during periods of high ED workload, there may be a tendency to prioritise rapid processing over careful assessment, potentially leading to referrals of patients who do not require specialist intervention [16].

Our closed-loop audit demonstrated clear and measurable improvements following the introduction of an acute abdominal pain pathway incorporating early CT imaging. In the second cycle, the proportion of patients referred without blood investigations fell significantly from 39.5% to 4.2% (p=0.001), and those without preliminary treatment declined from 21.9% to 6.9% (p=0.013). Notably, the number of inappropriate referrals decreased significantly from 27.9% in the first cycle to 9.7% in the second (p=0.004), highlighting the pathway's effectiveness in improving diagnostic accuracy, streamlining referral practices and enhancing patient flow. The findings of the present study demonstrate that surgical referral pathways incorporating an early CT scan at their core significantly reduce inappropriate referrals to the wrong speciality. For the surgical team, this translates into less time spent on avoidable consultations and negotiating with other specialities better equipped to manage the patient's condition. Consequently, this not only rationalises the workload but also reduces the likelihood of mismanagement or suboptimal care, thereby minimising the risk of adverse outcomes.

These findings are consistent with existing literature demonstrating that early CT scanning improves diagnostic accuracy, reduces unnecessary admissions and lowers the rate of inappropriate surgical referrals. A study by Weir-McCall et al. involving 196 patients found that the early utilisation of a CT scan increases early diagnosis and can be used as a valuable adjunct for the assessment of the acute abdomen [13]. Rosen et al. evaluated 536 consecutive patients with abdominal pain and demonstrated that a CT scan had the most significant influence on hospital admission and surgical management, thereby reinforcing the value of CT in the emergency setting [14]. Our findings add to this, as the early use of the CT scan in the acute abdominal pathway led to a significant reduction in the number of inappropriate referrals. It also improved the discrepancy in ED and GS assessment of the patients. This supports the growing body of evidence that early CT utilisation enhances diagnostic accuracy, facilitates appropriate speciality referral and ultimately improves patient flow and outcomes.

For patients, these improvements result in a much shorter waiting time and more timely access to the appropriate speciality for the optimal management of their condition. From a system perspective, this enhances NHS efficiency by reducing unnecessary investigations and duplicate testing, thereby lowering costs and leading to improved overall quality of care. Importantly, the intervention described here is readily generalisable and could be implemented across multiple hospitals and trusts. Widespread adoption would minimise time spent on avoidable consultations and patient reviews, enabling a prompt response to appropriate referrals. In addition to optimising resource utilisation in the short term, such changes are likely to improve working conditions and staff satisfaction over time, ultimately enhancing productivity and the quality of patient care.

Our study has a few limitations. It was a single-centre study, which may limit the applicability of the findings to other institutions with different referral patterns, available resources such as round-the-clock CT scan or local pathways. Both audit cycles were conducted over a relatively short period of one month each, which may not have fully captured seasonal variations in case load, staffing issues or pressures on the ED. We also did not explore the timing of referrals, for example, whether inappropriate referrals were more common around staff changeover times, when there may be greater pressure to 'clear the board' and expedite the patient flow. Our definition of 'inappropriate referral' was based on discordance between the initial referral diagnosis and the final surgical assessment diagnosis and onward referrals to other specialities. While this may reflect everyday clinical practice, there is a potential for interobserver variability and subjectivity. Our sample size was modest; hence, there is a chance of missing more subtle but clinically relevant differences. Appropriate referrals to a speciality are an important quality metric for healthcare facilities; our audit did not look at the patient-centred outcomes such as time to definitive treatment, the length of hospital stay, morbidity or patient satisfaction. Future work on this subject should include longer data collection periods across multiple centres with a larger sample size and the timing of referrals, along with the evaluation of patient outcomes. This would help strengthen the evidence for the acute abdominal pain pathway and guide further improvements in referral practice.

## Conclusions

This study demonstrates that the implementation of an acute abdominal pain pathway incorporating early CT imaging has the potential to significantly improve the appropriateness of surgical referrals from the ED, but there is a need for an extensive multicentre study to confirm the generalisability.

With the introduction of the acute abdominal pain pathway, in our setup, there was a significant reduction in inappropriate patient referrals, along with a decrease in the number of patients not receiving correct preliminary treatment, highlighting the effectiveness and thereby streamlining patient flow. The early use of CT scans helps in facilitating timely and accurate speciality triage, reduces unnecessary admissions and optimises resource allocation, thereby improving both patient outcomes and healthcare efficiency. These findings are consistent with the existing literature and underline the potential for the widespread adoption of early CT-based pathways across hospitals and trusts to enhance service delivery, reduce avoidable workload and maintain high standards of care. Ultimately, targeted interventions such as this provide a pragmatic approach to addressing systemic pressures while safeguarding patient safety and improving staff efficiency.
